# Stereo-Selective Pharmacokinetics of Ilimaquinone Epimers Extracted from a Marine Sponge in Rats

**DOI:** 10.3390/md17030171

**Published:** 2019-03-17

**Authors:** Heebin Son, Keumhan Noh, InWha Park, MinKyun Na, Sangtaek Oh, Beom Soo Shin, Wonku Kang

**Affiliations:** 1 College of Pharmacy, Chung-Ang University, Seoul 06974, Korea; amybin2@naver.com; 2 Department of Pharmaceutical Sciences, Leslie Dan Faculty of Pharmacy, University of Toronto, 144 College Street, Toronto, ON M5S 3M2, Canada; keumhan.noh@utoronto.ca; 3 College of Pharmacy, Chungnam National University, Daejeon 34134, Korea; inwha129@naver.com (I.P.); mkna@cnu.ac.kr (M.N.); 4 Department of Bio and Fermentation Convergence Technology, BK21 PLUS Program, Kookmin University, Seoul 02707, Korea; ohsa@kookmin.ac.kr; 5 School of Pharmacy, Sungkyunkwan University, Suwon 16419, Korea

**Keywords:** ilimaquinone, epi-ilimaquinone, stereo-selective pharmacokinetics, HPLC-MS/MS, rat

## Abstract

An ilimquinone (IQ) mixture isolated from *Hippiospongia metachromia*, consisting of IQ and epi-ilimaquinone (epi-IQ), exerts anti-HIV, anti-microbial, anti-inflammatory, and anti-cancer effects. An HPLC-MS/MS method was developed for simultaneous determination of the two epimers in rat plasma, separating them using a biphenyl column. Ascorbic acid is added during the sample preparation to ensure the stability of both isomers. The plasma concentrations of the isomers were monitored following intravenous and oral administration of the IQ mixture in rats as well as the individual epimers that were separately orally administered. Compare to IQ, epi-IQ was much more stable in rat plasma, likely due to its configurations of decalin. Both substances decayed in more than bi-exponential pattern, with an elimination rate constant of 1.2 h^−1^ for IQ and 1.7 h^−1^ for epi-IQ. The epi-IQ was distributed more widely than IQ by about two-fold. Consequently, the clearance of epi-IQ was greater than that of IQ by about three-fold. The oral absolute bioavailability for IQ was 38%, and, that for epi-IQ, was 13%. Although the systemic exposure of IQ was greater than that of epi-IQ by ~8.7-fold, the clearance of each isomer was similar when administered either orally or intravenously, when normalized for bioavailability. The stereo-specific behavior of the isomers appears to originate from differences in both their tissue distribution and gastrointestinal permeability.

## 1. Introduction

Ilimaquinone (IQ) is isolated from *Hippiospongia metachromia* [1], which is a marine sponge. This substance exhibits several pharmacological effects, including antiviral action against human immune-sufficient virus [2], antibacterial properties against *Staphylococcus aureus* [3], anti-cancer activity for various types of cancer cells [4,5,6,7,8], and inhibitory activity against nitrogen oxide (NO) production in lipopolysaccharide-stimulated in BV2 microglia cells [9].

**Figure 1:**
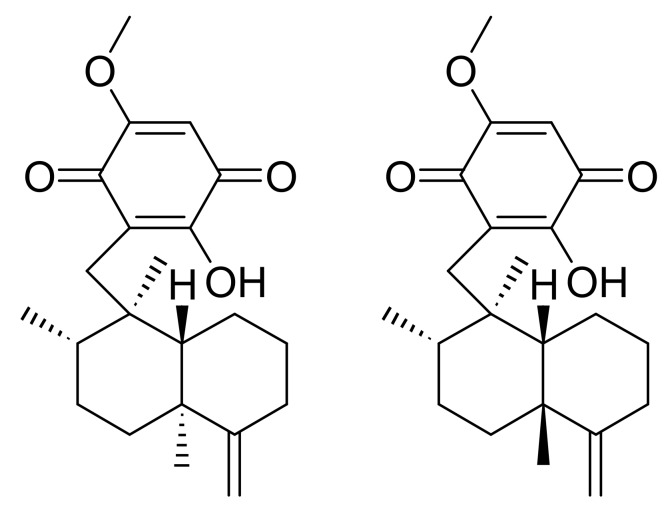
Chemical structures of ilimaquinone (IQ; **left**) and epi-ilimaquinone (epi-IQ; **right**).

In nature, the substance mainly exists as a mixture of epimers, IQ, and epi-ilimaquinone (epi-IQ), at a ratio of 2:1 (Figure 1). However, both isomers have revealed to contribute equally to decreasing the level of β-catenin, which suppresses the Wnt/β-catenin pathway [6]. Their pharmacokinetic behaviors are poorly characterized. The pharmacokinetic profiles of both epimers can be useful not only to understand their pharmacological consequences, but also to create strategies for optimal dosing regimens. In addition, the interactions in between epimers and the epimeric conversion in vivo would also be interesting, when the extract as a mixture of both epimers is administered. A recent report described a novel analytical method for determining IQ in rat plasma using high-performance liquid chromatography coupled to tandem mass spectrometry (HPLC-MS/MS). It was also used in a pharmacokinetic study following per oral (p.o.) administration of IQ [10]. Because IQ is unstable in rat plasma, ascorbic acid was added to the plasma during sample preparation to ensure the chemical stability of the quinone moiety of IQ [11]. IQ decayed mono-exponentially with a half-life of 1.2 h after peaking at about 2.5 h.

This study was designed to investigate the stereo-selective pharmacokinetic properties of IQ and epi-IQ, which is extracted from the marine sponge in rats. The epimers were separated with good resolution using a stationary column providing a hydrophobic interaction and the stability of epi-IQ was evaluated prior to the animal study. The mixture was given to rats by both the p.o. and intravenous (i.v.) routes. Individual epimers were separately given orally to clarify the drug-drug interactions between the epimers and to monitor the epimeric biotransformation in vivo.

## 2. Results

### 2.1. Separation and Quantification of IQ and epi-IQ

**Figure 2:**
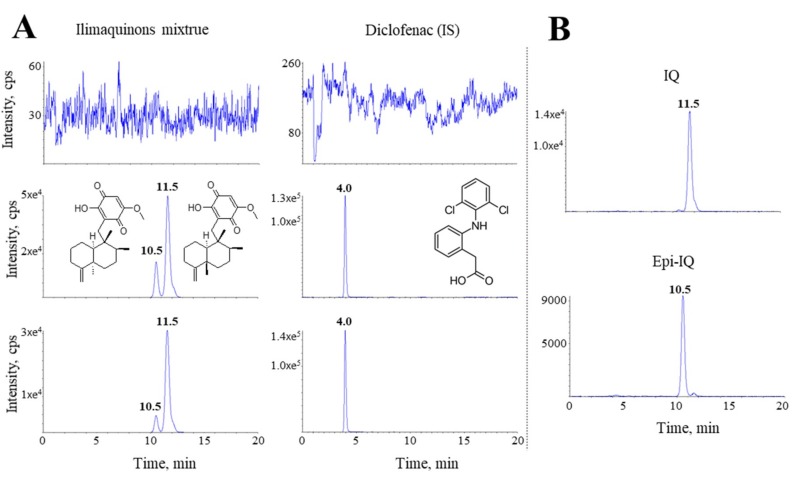
Chromatograms of IQ mixture in plasma (**A**) and each isomer in methanol (**B**). (**A**) Top, double-blank plasma. Middle, plasma spiked with 500 ng/mL IQ mixture (333 ng/mL of IQ and 167 ng/mL of epi-IQ) and 100 ng/mL of the IS. Bottom, a real plasma sample containing 324 ng/mL of IQ and 58 ng/mL of epi-IQ. The monitored mass transitions for IQs and the IS were *m*/*z* 357.0→151.0 and 296.1→251.7, respectively.

In the present method, epi-IQ and IQ elute at 10.5 and 11.5 min, respectively, with good resolution (Figure 2). No endogenous interference is evident at the elution times of either substance. The calibration curves used ensured a reliable response for the two compounds. The ratios of the peak areas of IQ and epi-IQ relative to that of the IS are correlated with the corresponding plasma concentrations and good linearity is evident (*r*^2^ > 0.997 for IQ, *r*^2^ > 0.996 for epi-IQ). The quantitation limits for IQ and epi-IQ were 3 and 2 ng/mL, respectively, at a signal-to-noise (S/N) ratio of 5.

### 2.2. Assay Validation and Stability of epi-IQ in Rat Plasma

**Figure 3:**
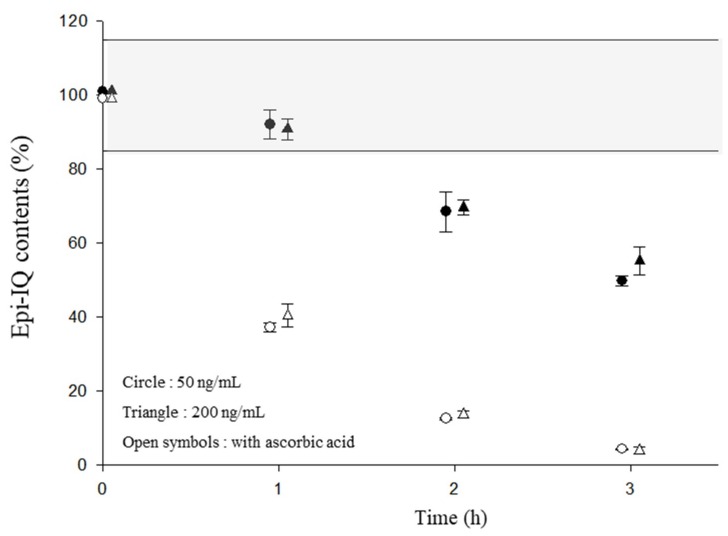
Stability of epi-IQ in rat plasma. The shaded zone indicates the acceptable range (85% to 115%).

The plasma calibration curves provided a reliable response with a good linearity (*r*^2^ > 0.998) and the limit of detection was 1 ng/mL for epi-IQ at a signal-to-noise (S/N) ratio of 3. The method is accurate and precise for the determination of epi-IQ in rat plasma, as shown in Table 1. IQ is stable for up to 3 h at room temperature in the presence of ascorbic acid [10]. However, the method revealed that epi-IQ showed endurance of only 1 h in rat plasma despite the presence of the ascorbic acid stabilizer (Figure 3). Thus, the stabilizer (5 mg/mL) should be added during sample preparation to guarantee both freeze-thaw and long-term stability. No dilution effect for epi-IQ up to 10 μg/mL was found by means of a 10-fold dilution with blank rat plasma.

### 2.3. Stereo-Selective Pharmacokinetics of IQ Epimers

**Figure 4:**
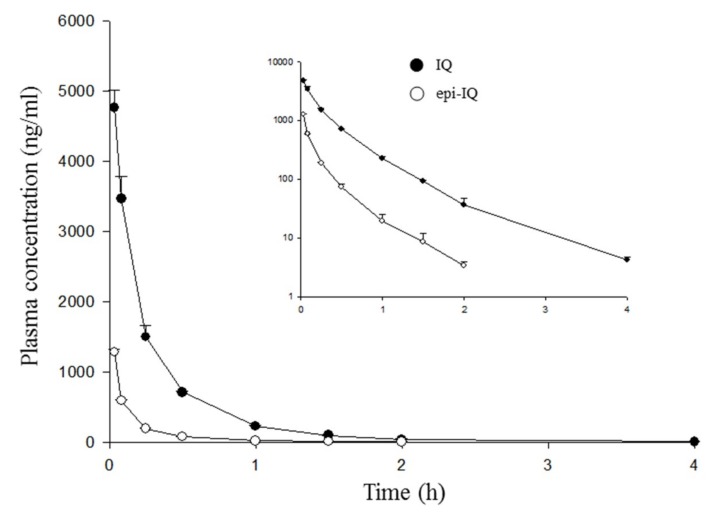
Time course of plasma IQ and epi-IQ concentrations following a single intravenous injection of the IQ mixture 3 mg/kg (2 mg/kg of IQ and 1 mg/kg of epi-IQ) in rats. The insert shows semi-logarithmic graphs. Each point represents the mean ± SD (*n* = 5).

The validated HPLC-MS/MS method was used to measure IQ and epi-IQ simultaneously in rat plasma after intravenous or oral administration of the IQ mixture or individual epimers. A purified IQ mixture extracted from the marine sponge was i.v.-administered at 3 mg/kg (2 mg/kg of IQ, 1 mg/kg of epi-IQ). Time courses of plasma concentrations of both epimers are shown in Figure 4. Pharmacokinetic parameters are listed in Table 2.

Both substances decayed in a more than bi-exponential pattern, with the elimination rate constants of 1.2 h^−1^ for IQ and 1.7 h^−1^ for epi-IQ (*p* < 0.05). The initial volume of distribution (V_c_) calculated from the concentrations extrapolated at time 0 h was 0.36 ± 0.01 L/kg for IQ and 0.58 ± 0.02 L/kg for epi-IQ (*p* < 0.01), and the volume of distribution at a steady state was estimated to be 1.14 ± 0.08 and 2.43 ± 0.22 L/kg (*p* < 0.005), respectively. Although epi-IQ was distributed more widely than IQ by about two-fold, the elimination rate constant of epi-IQ at the terminal phase (1.7 h^−1^) was greater than that of IQ by 41%. Consequently, the clearance of epi-IQ is greater than that of IQ by about three-fold (*p* < 0.005).

**Figure 5:**
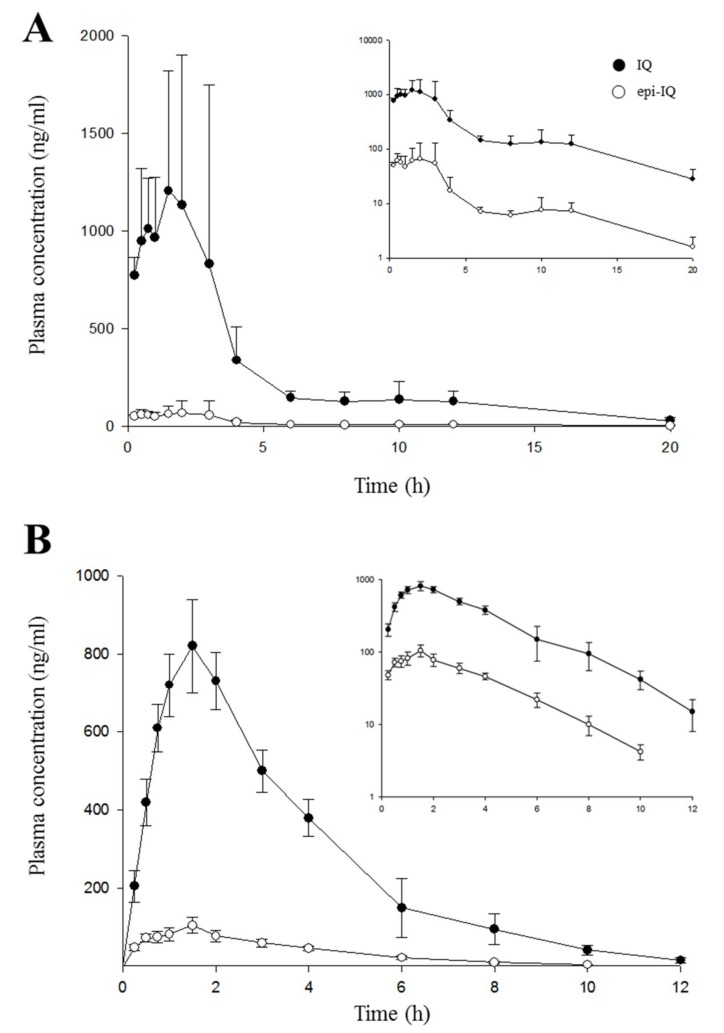
Time courses of plasma IQ and epi-IQ concentrations following oral administration of a mixture at 30 mg/kg (20 mg/kg of IQ and 10 mg/kg of epi-IQ) (**A**) or individual epimers (**B**) at 10 mg/kg in rats. Inserts represent semi-logarithmic graphs and each point represents the mean ± SD (*n* = 5).

Figure 5 illustrates the plasma IQ and epi-IQ concentrations over time following oral administration of the mixture 30 mg/kg (20 mg/kg of IQ and 10 mg/kg of epi-IQ) (A) and the individual epimers (B) at 10 mg/kg. In the time course, the plasma epi-IQ concentrations are shifted downward from those of IQ with each showing a similar profile. Double peaks were observed when the mixture was given orally. After reaching peaks of 1.29 μg/mL for IQ and 0.11 μg/mL for epi-IQ at around 2 h, the plasma concentrations decreased to 0.21 μg/mL for IQ and 0.07 μg/mL for epi-IQ by about 8 h. Second peaks about 10-fold smaller than the first ones appeared at 10~12 h and decayed with a terminal half-life of about 4 h. The systemic exposure of IQ was greater, by ~8.7-fold, than that of epi-IQ, considering the dose difference, and clearance of the latter was greater than that of the former. The absolute oral bioavailability was 38% for IQ and 13% for epi-IQ. There was no difference in the clearance of the epimers compared to that observed after intravenous administration, i.e., after normalization by the bioavailability.

The double-peak phenomenon was not observed when the epimers were given individually by the oral route (Figure 5B). The time course of the plasma epi-IQ concentrations showed a downward shift with a profile similar to that of IQ, and similar to the results observed when both epimers were given simultaneously. The C_max_ (0.94 ± 0.19 μg/mL) and AUC (3.39 ± 0.61 μg·h/mL) of IQ were greater than those of epi-IQ by 8.5-fold and 10.0-fold, respectively. The clearance of epi-IQ was 10-fold greater than that of IQ, whereas the elimination rate constant (0.6 h^−1^) and half-life (1.2 h) of each isomer were the same. The clearance, as normalized by the bioavailability, of each epimer was similar to that obtained following both intravenous and oral administration of the mixture of epimers. The elimination rate constant (0.6 h^−1^) of each epimer was 3-fold (*p* < 0.05) larger than when the epimers that were given as a mixture, and the half-life was accordingly shorter. This is attributed to the double-peak phenomenon. No adverse effect was found at the current dosages of IQ, epi-IQ, and the mixture.

## 3. Discussion & Conclusions

A validated quantitative determination method is a prerequisite for obtaining accurate plasma concentrations. In this work, both IQ and epi-IQ were accurately and precisely determined in rat plasma using a single method for the first time. Optimum pre-treatment procedures and storage conditions for the plasma sample are presented that ensure the stability of both substances. The method has been successfully used to characterize the stereo-selective pharmacokinetics of IQ and epi-IQ in rats.

**Figure 6:**
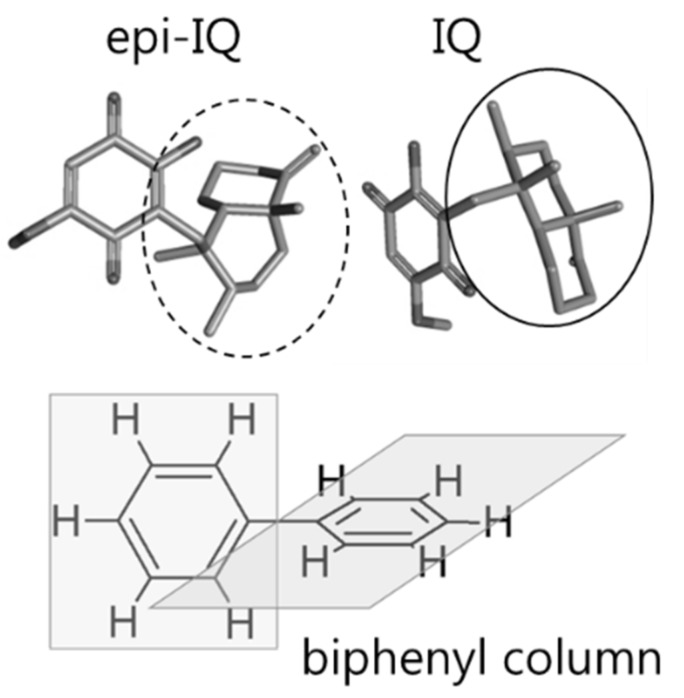
Three-dimensional structures of IQ, epi-IQ, and the reactive group of the biphenyl column stationary phase. Straight and dashed ovals indicate the decalin moieties of IQ and epi-IQ, respectively.

When extracted from the marine sponge *Hippiospongia metachromia*, IQ, 3-[[(1R,2,4a**S**,8aS)-1,2,4a-trimethyl-5-methylidene-3,4,6,7,8,8a-hexahydro-2H-naphthalen-1-yl]methyl]-2-hydroxy-5-methoxycyclohexa-2,5-diene-1,4-dione, naturally exists as a mixture with epi-I. The chemical difference of the epimers resides at the carbon 4a position of the decalin moiety, with the absolute configuration of the carbon of IQ representing, *S*, and that of epi-IQ representing *R*. Thus, a chromatographic separation was conducted using hydrophobic interactions between decalin and a column with a biphenyl stationary phase (Figure 6). The plain *trans*-decalin structure (straight oval) of IQ likely interacts with the biphenyl group for longer than the bent *cis*-decalin of epi-IQ (dashed oval), which results in delayed elution of the former substance and the clear resolution of both epimers. Because ring-flipping cannot occur in the *trans*-decalin structure, IQ represents a structure with less ring strain and more stability in terms of the energy state than epi-IQ. This likely explains the observation that IQ exists in greater quantities in nature than epi-IQ.

Compare to IQ, epi-IQ is less stable in rat plasma. The sample preparation procedure for epi-IQ at room temperature must be conducted within 1 h even in the presence of ascorbic acid, and the stabilizer should be added to ensure the stability of the compound during the frozen storage. Even though whether the instability stems from the quinone and/or decalin moiety or from the linkage between the moieties was not investigated, it is speculated that the instability originates from the former structure rather than the latter. The differences in stability of each epimer may be due to the different configurations of decalin; i.e., the plain structure of decalin in IQ may provide more steric hindrance to resist chemical change due to a possible enzymatic attack than the bent decalin present in epi-IQ (Figure 6).

Stereo-selective pharmacokinetic studies are open conducted because most drugs are administered as a mixture of stereoisomers [12,13,14,15,16]. The effects of stereo-specificity on the pharmacokinetics of drugs may be due to stereo-selective metabolism and/or distribution, including absorption. Paclitaxel [12] and 25-methoxyl-dammarane-3β,12β,20(R/S)-triol [13] are good examples of drugs that show enantio-selective metabolism related to their configurations at the 7-carbon and 20-carbon positions, respectively. The systemic exposure of 22*R*-budesonide is six-fold less than that of the 22*S* form due to their enantio-specific distribution [14]. The *R*-epimers of the ginsenoside analogues Rh2 and Rg3 are poorly absorbed compared to the *S*-isomers, possibly due to their lower membrane permeability and extensive intestinal biotransformation [15]. (+)-*alpha-*Benidipine also shows two-fold greater absorption than the (−)-*alpha* isomer. However, the forms show no differences in elimination rates [16]. In contrast to the examples with the chiral center at a non-cyclic carbon presented above, rhynchophylline contains a chiral center at the carbon linking two five-membered rings. Its oral bioavailability is much greater (~9-fold) than that of isorhynchophylline. However, there was no difference in pharmacokinetic parameters of these epimers following intravenous administration [17], which indicates that the stereospecific first-pass metabolism is the dominant factor in the difference. Stereo-selective pharmacokinetics of a substance with decalin moiety participating in two carbons in a bi-cyclic ring have been reported only rarely. Although Dill et al. reported the results of a toxicokinetic study of decalin in murine animals, stereo-specificity was not investigated [18].

To the best of our knowledge, this is the first investigation of the stereospecific pharmacokinetics of IQ epimers. The dominance of systemic exposure of IQ over that of epi-IQ seems to stem from the differences not only in their infiltration into tissues, but also in their permeability through the gastro-intestinal tracts rather than metabolism. The time courses of the plasma concentrations of the epimers were shifted but with a similar profile. There was no difference in the terminal half-lives of the epimers following oral administration. Although there appears to be a change in elimination rates after intravenous administration, the observed change might be due to analytical variability in the concentrations measured at a low level in plasma. The overall patterns of both epimers in systemic circulation (Figure 4) appear to be in accordance with those following oral dosing (Figure 5). The lower stability of epi-IQ compared to IQ in the biological samples could also have had an effect, but it should not have a larger influence in vivo considering the identical half-lives of both epimers.

The absolute oral bioavailability of both isomers is estimated to be about 30%, factoring in the composition of the mixture in nature. Recently, because the isomers have been reported to exert equal pharmacodynamic actions [6], it has become apparent that they may not need to be separated. In many drugs in which one isomer exerts beneficial effects, the other has undesirable actions such as toxicities. Therefore, separation by means of chromatography and/or specific synthesis is needed in some cases.

Double peaks were only observed when the mixture was given orally, which lengthened the terminal half-lives of both epimers significantly (*p* < 0.05) even though this had no effect on the systemic exposure. This phenomenon should be investigated further in spite of minor pharmacological consequences expected.

No interaction between the isomers was found. The total clearance for each epimer was almost the same whether administered individually or simultaneously. Although the maximum concentration of IQ, when given orally alone, was slightly higher than that following oral administration of the mixture, there was no statistical difference. In addition, no epimeric conversion was observed. No peak of the counterpart isomer appeared when either epimer was given individually.

Doses for pharmacokinetic investigations could be selected based on the pharmacological and/or toxicological studies. Although, to date, 61 research studies related to IQ can be found in Pubmed, no animal experiment has been published except for this recent work [10]. The doses should be low enough to characterize the pharmacokinetic profiles as long as a determination method provides enough sensitivity because a high dose can provide improper saturable pharamcokinetic data. One to 10 and 10~100 mg/kg doses are generally accepted for pharmacokinetic studies following intravenous and oral administrations, respectively.

In conclusion, a sensitive HPLC-MS/MS method was developed for the simultaneous determination of IQ and epi-IQ in rat plasma. The absolute oral bioavailability of the mixture was about 30% (38% for IQ and 13% for epi-IQ). The systemic exposure of IQ is 10 times greater than that of epi-IQ. The clearance was three-fold higher considering the oral absorption. The stereo-specific behavior of the epimers appears to originate from differences in both tissue distribution and gastrointestinal permeability.

## 4. Materials and Methods

### 4.1. Chemicals and Reagents

The IQ epimers were extracted and separated from *Hippiospongia metachromia*. Diclofenac (internal standard, IS), formic acid, and ascorbic acid were purchased from Sigma-Aldrich (Seoul, Korea). Methanol and acetonitrile were obtained from Burdick & Jackson (Muskegon, MI, USA). All other chemicals and solvents were of the highest analytical grade available.

### 4.2. Preparation of Standards and Quality Controls

IQ, epi-IQ, and the IS were separately dissolved in methanol at 1 mg/mL, and serially diluted with the same solvent. Then, 10 μL of the diluted solutions of each IQ epimer was added to 80 μL of blank rat plasma to obtain final concentrations at 2, 5, 10, 50, 100, 500, and 1000 ng/mL for each epimer. Using linear regression, calibration graphs were derived from the ratios between the peak areas of each epimer with those of the IS.

Quality control (QC) samples were prepared in 90 μL of drug-free plasma by adding 10 μL of serially diluted epi-IQ solutions to obtain concentrations at the lower limit of quantification (LLOQ, 2 ng/mL), and at low (10 ng/mL), intermediate (100 ng/mL), and high (800 ng/mL) concentrations. These samples were used to evaluate the intra-day and inter-day precision and accuracy of the epi-IQ assay. Validation of the IQ assay was carried out previously [10].

### 4.3. Characterization of Product Ions Using MS/MS

Solutions of IQ, epi-IQ, and the IS in methanol (10 ng/mL) were individually infused into the mass spectrometer at a flow rate of 10 μL/min to characterize the precursor and the product ions. Precursor ions and the fragmentation patterns were monitored in a negative-ion mode. Major peaks in the MS/MS scan were used to quantify the substances.

### 4.4. Analytical System

Plasma concentrations of IQ and epi-IQ were quantified using an API 4000 LC/MS/MS system (AB SCIEX, Framingham, MA, USA) equipped with an electrospray ionization interface. The compounds were separated on a Kinetex biphenyl column (100 × 2.1 mm internal diameter, 100-Å pore size, 2.6-μm particle size, Phenomenex, Torrance, CA, USA) in a mobile phase of water:acetonitrile mixture at a 1:3 (*v*/*v*) ratio and also including 0.1% formic acid. The column was heated at 30 °C and the mobile phase was delivered at a flow rate of 0.2 mL/min using an HP 1100 series pump (Agilent, Wilmington, DE, USA).

The turbo ion spray interface was operated at 4500 V at 450 °C. Both the precursor and product ions of IQ, epi-IQ, and the IS appeared predominantly as deprotonated ions [M − H]^−^, at an *m*/*z* of 357.0 for both epimers and 296.1 for the IS. After collision with nitrogen in Q2, the product ions were scanned in Q3 at an *m*/*z* of 151.0 (declustering potential, −50 eV, collision energy, −54 eV, dwell time, 150 ms) for the IQ epimers and 251.7 (declustering potential, −65 eV, collision energy, −18 eV, dwell time, 150 ms) for the IS. The deprotonated precursor ions and related product ions were quantified by selective reaction monitoring using the peak area ratios of each substance. The analytical data were processed using Analyst software (ver. 1.5.2, Applied Biosystems, Foster City, CA, USA).

### 4.5. Plasma Sample Preparation

First, 90 μL of an IS solution (100 ng/mL in methanol) was added to 30 μL of plasma, after which the mixture was shaken vigorously for 10 s. After centrifugation at 17,000 rpm for 10 min at 4 °C, 5 μL of the supernatant was injected into the column.

### 4.6. Method Validation and Stability of epi-IQ

The intra-day and inter-day precision and accuracy of the assay were tested with five replicates of four QC samples (LLOQ, low, medium, and high), with concentrations determined using calibration curves. Acceptable criteria for precision and accuracy are results within ±15%. However, those for the LLOQ should be within ±20%. The dilution effect of IQ and epi-IQ for samples at 5 and 10 μg/mL was examined using a 10-fold dilution with drug-free plasma.

To ensure the stability of epi-IQ in rat plasma, ascorbic acid was added at a final concentration of 5 mg/mL to plasma samples containing 50 or 200 ng/mL epi-IQ. Room temperature and long-term stability were assessed for up to 2 h and 4 weeks at −70 °C, respectively. Three freeze-thaw cycles (−70 °C to room temperature) of plasma samples and storage of the extracts for 24 h at 4 °C were used to evaluate the freeze-thaw and post-extraction stability, respectively. The stability of these samples was analyzed by comparing results with those of samples freshly prepared in the presence of ascorbic acid. Differences less than ±15% were deemed acceptable.

### 4.7. Animal Experiments

Twenty male Sprague-Dawley rats were divided into four groups (five rats in each group): mixture p.o. and i.v. groups, and individual IQ/epi-IQ p.o. groups. IQ and epi-IQ were suspended in corn oil for oral administration, and dissolved in a mixture of normal saline, dimethylacetamide, polyethylene glycol 400, and dimethylsulfoxide (3.5:3:3:0.5) for intravenous dosing.

The mixture of both epimers was administered orally and intravenously at 30 mg/kg (20 mg/kg for IQ, 10 mg/kg for epi-IQ) and 3 mg/kg (2 mg/kg for IQ, 1 mg/kg for epi-IQ), respectively. Heparinized blood samples (100 μL) were serially collected from subclavian veins at 0, 2, 5, 15, and 30 min, and 1, 1.5, 2, 4, 6, 8, and 10 h for intravenous administration, and at 0, 0.25, 0.5, 0.75, 1, 1.5, 2, 3, 4, 6, 8, 10, 12, and 20 h for oral dosing.

All samples were stored in tubes with ascorbic acid added to 5 mg/mL. After centrifugation at 17,000 rpm for 10 min, plasma samples (30 μL) were prepared by immediately adding 90 μL of methanol containing the IS. The Institutional Animal Care and Use Committee at Chung-Ang University (No. 201900025) approved this study.

### 4.8. Data Analysis and Statistics

Time courses of plasma IQ and epi-IQ concentrations were used to calculate the pharmacokinetic parameters: peak concentration (C_max_) and time to C_max_ (T_max_) were directly obtained from the individual time courses. The elimination rate constant (k) was obtained by linear regression from the log-transformed plasma concentrations at the terminal phase. The area under the plasma concentration time curve (AUC_t_) was calculated using the trapezoidal rule and concentration at the last sampling time (C_last_)/k was added to obtain the AUC to infinite (AUC_inf_). Clearance was calculated by dose/AUC_inf_, and the absolute bioavailability of each epimer was estimated using the equation AUC_inf,p.o._ × Dose_i.v._/(AUC_inf,i.v._ × Dose_p.o._).

All data are presented as mean ± standard deviation. The pharmacokinetic parameters were statistically compared using Student’s *t*-test and statistical significance was taken as *p* < 0.05.

## Figures and Tables

**Table 1:** Accuracy, precision, and stability of epi-IQ in rat plasma.

Concentration (ng/mL)	Intra-Day	Inter-Day	Conditions for Stability Test	50 ng/mL	200 ng/mL
2	101.4 ± 4.0 (4.0) ^1^	100.0 ± 4.0 (4.0)	Room temperature for 1 h	92.1 ± 3.9 ^2^	90.7 ± 2.8
10	103.9 ± 3.2 (3.1)	109.1 ± 5.5 (5.0)	3 freeze-thaw cycles	94.7 ± 2.3	95.5 ± 3.6
100	107.7 ± 3.8 (3.5)	99.3 ± 1.4 (1.4)	Post-extraction at 4 °C for 24 h	102.9 ± 3.8	100.0 ± 0.4
800	104.3 ± 3.1 (3.0)	91.7 ± 3.2 (3.5)	−70 °C for 4 weeks	95.7 ± 6.8	94.0 ± 4.2

^1^ mean accuracy % ± s.d. (relative standard deviation, %), ^2^ mean % ± s.d.

**Table 2:** Pharmacokinetic parameters of IQ and epi-IQ.

Parameter	Mixture i.v.		Mixture p.o.		Individual p.o.	
	IQ (2 mg/kg)	epi-IQ (1 mg/kg)	IQ (20 mg/kg)	epi-IQ (10 mg/kg)	IQ (10 mg/kg)	epi-IQ (10 mg/kg)
C_max_ (μg/mL)	-	-	1.29 ± 0.33	0.11 ± 0.03	0.94 ± 0.19	0.11 ± 0.02
T_max_ (h)	-	-	1.3 ± 0.8	1.7 ± 1.3	2.5 ± 1.3	1.7 ± 0.3
k (h^−1^)	1.2 ± 0.0	1.7 ± 0.1 ^†^	0.2 ± 0.1	0.2 ± 0.1	0.6 ± 0.1 *	0.6 ± 0.1 *
t_1/2_ (h)	0.6 ± 0.0	0.4 ± 0.0 ^†^	3.8 ± 0.9	3.9 ± 1.1	1.2 ± 0.3 *	1.2 ± 0.3 *
AUC_inf_ (μg·h/mL)	1.46 ± 0.11	0.24 ± 0.03 ^††^	5.55 ± 1.19	0.32 ± 0.12	3.39 ± 0.61	0.34 ± 0.05
Cl (L/h/kg)	1.37 ± 0.10	4.20 ± 0.53 ^†††^	3.64 ± 0.77 (1.38 ± 0.29) ^1^	30.25 ± 11.3 (3.93 ± 1.47) ^1^	2.95 ± 0.53 (1.12 ± 0.20) ^1^	29.4 ± 4.32 (3.82 ± 0.56) ^1^
V_c_ (L/kg)	0.36 ± 0.01	0.58 ± 0.02 ^††^	-	-	-	-
V_ss_ (L/kg)	1.14 ± 0.08	2.43 ± 0.22 ^†††^	-	-	-	-
F (%)			38	13	-	-

^†^*p* < 0.05, ^††^ *p* < 0.01, ^†††^ *p* < 0.005 compare to IQ. * *p* < 0.01 compare to mixture p.o. ^1^ Clearance normalized by F.
